# Acknowledgment to the Reviewers of *Pediatric Reports* in 2022

**DOI:** 10.3390/pediatric15010007

**Published:** 2023-01-17

**Authors:** Pediatric Reports Editorial Office

**Affiliations:** MDPI AG, St. Alban-Anlage 66, 4052 Basel, Switzerland

High-quality academic publishing is built on rigorous peer review. *Pediatric Reports* was able to uphold its high standards for published papers due to the outstanding efforts of our reviewers. Thanks to the efforts of our reviewers in 2022, the median time to first decision was 31 days and the median time to publication was 56 days. Regardless of whether the articles they examined were ultimately published, the editors would like to express their appreciation and thank the following reviewers for the time and dedication that they have shown *Pediatric Reports*:
Abdullah TolaymatKeisuke JimboAdebayo AdeyinkaKeita TeruiAhmed H. MekkawyKejd BiciAlejandro Martínez-RodríguezKhalim KhujamatovAleksandra MorkaKlaus RoseAlessandra VerzelloniKristijan SkokAlessia RenziKumar DigvijayAlexandrina S. NikovaKundlik GadhaveAlher Mauricio HernandezLacramioara OchiuzAmanda RamosLaura CannavòAndrea VitaliLeonardo CesanelliAnna BorrelliLingpeng ShanAnna Maria CostaLorena AiraghiAnna MrzljakMaicol ManciniAnna PiotrowskaMangla SoodAntonio BiondiManish TripathiAntonio GattoManju M. ChandraArnaud LombardMarcin TkaczykAssunta TorneselloMarco FerrariAurelio LunaMargarita RomanenkoBarbara CuomoMargherita ScapaticciBarbara Medoff-CooperMaria KalafatiBart RuttemanMarina CuttiniBianca N. JacksonMark EsserCarina De Fátima RodriguesMarko BaškovićCarina Pinheiro-AraujoMarzia CottiniCarla Di StefanoMazur-Melewska KatarzynaCarlos Martin LlorenteMd MohiuddinCarole A. Samango‐SprouseMd. Hakimul HaqueCecilia Beatrice ChighizolaMichele SantoroChi-Hui ChengMinhhuyen T. NguyenChina NaganoMoises Alejandro Alatorre-JimenezChristian SiatkaMuzamil WantChristoph StrumannNadia NajafiChristophe GoubauNagasuryaprasad KotikalapudiChristos KourekNaoro TanakaCiesla MaciejNicola ZampieriCigdem I. AkmanNils JaekelClaudio GiraudoOmar AlibrahimCoralie GandreOrjena ŽajaCristian TieranuPalanikumar BalasundaramCristina Floriana PanăPaul DrewCristina SecosanPaul SchoenhagenDalia GobbiPaula Fernández-PiresDebipreeta BhowmikPetar AvramovskiDeftereos SavasPhilip LiDhara SharmaPierre Simon RöhrlichDian-Fu ChangPin WangDilipkumar R. PatelPreeti ChauhanDimitra DimopoulouRalph D. HectorDimitrios KaragiannakisRaquel Leirós-RodríguezDong Keon YonRichard W. NaylorEl Hadji M. DioumRobert RoghairEleni VergadiRoger C. HoElizabeth KertowidjojoRoksana MalakElżbieta PoniewierkaRozeta SokouEmmanouil KapetanakisRubén Arroyo FernándezEmmanuel RineauRuslan BohovykEnrico VallettaSachit AnandEvanthia BletsaSami M. Abd ElwahabFrances M. AlbaSaral DesaiFrancesca ColoneseScott T. McEwenFrancisco José García-MoroShannon Scott-VernagliaFrank Blanco-PerezShenqin YaoFrank E. NelsonSheree YorkGeorge ImatakaSilvia AkutsuGerald FerrettiSilvia D’AgostinoGiacomo SteraStefan Holland-CunzGizem KeceliStefania PaduanoGusztav BeltekiStephanie C. DeLucaHang XingSteve GrahamHeather Leduc-PessahSudip ShethHimanshu BatraSupriya ChakrabortyHong FangTalida Georgiana CutHuihui HuangToru IwasaIlhak LeeToshihiro KawaeJacques De MouzonVijay IndukuriJan BroggerVikash AgrawalJeremie D. OliverVittoria RaimondiJesús CespónWalid ShalataJesús Martínez De La CalWei NiJesus S. JimenezWiktoria WojniczJiuann-Huey Ivy LinWooyeong ParkJohanna KostenzerXiphias ZhuJohn Griffith ToffalettiYashomati M. PatelJose Boix-OchoaYoichiro OdaJulie HuynhYu-Huei LiuKai Cheng HsuYuzhe LiuKashish MehraZenon PogorelićKathleen RyanŽivka DikaKazem NazariZorica Kauric-Klein


